# Carbon accretion in unthinned and thinned young-growth forest stands of the Alaskan perhumid coastal temperate rainforest

**DOI:** 10.1186/s13021-015-0035-4

**Published:** 2015-10-20

**Authors:** David V. D’Amore, Kiva L. Oken, Paul A. Herendeen, E. Ashley Steel, Paul E. Hennon

**Affiliations:** 1 U.S. Department of Agriculture, Forest Service, Pacific Northwest Research Station, Juneau Forestry Sciences Laboratory, 11175 Auke Lake Way, Juneau, AK 99801 USA; 2 Quantitative Ecology and Resource Management, University of Washington, Box 355020, Seattle, WA 98195 USA; 3 Graduate Degree Program in Ecology, Colorado State University, Fort Collins, CO 80523 USA; 4 U.S. Department of Agriculture, Forest Service, Pacific Northwest Research Station, 400 N 34th Street, Suite 201, Seattle, WA 98103 USA

**Keywords:** Carbon sequestration, Young-growth, Stand management, Ecosystem productivity, Natural resource management

## Abstract

**Background:**

Accounting for carbon gains and losses in young-growth forests is a key part of carbon assessments. A common silvicultural practice in young forests is thinning to increase the growth rate of residual trees. However, the effect of thinning on total stand carbon stock in these stands is uncertain. In this study we used data from 284 long-term growth and yield plots to quantify the carbon stock in unthinned and thinned young growth conifer stands in the Alaskan coastal temperate rainforest. We estimated carbon stocks and carbon accretion rates for three thinning treatments (basal area removal of 47, 60, and 73 %) and a no-thin treatment across a range of productivity classes and ages. We also accounted for the carbon content in dead trees to quantify the influence of both thinning and natural mortality in unthinned stands.

**Results:**

The total tree carbon stock in naturally-regenerating unthinned young-growth forests estimated as the asymptote of the accretion curve was 484 (±26) Mg C ha^−1^ for live and dead trees and 398 (±20) Mg C ha^−1^ for live trees only. The total tree carbon stock was reduced by 16, 26, and 39 % at stand age 40 y across the increasing range of basal area removal. Modeled linear carbon accretion rates of stands 40 years after treatment were not markedly different with increasing intensity of basal area removal from reference stand values of 4.45 Mg C ha^−1^ year^−1^to treatment stand values of 5.01, 4.83, and 4.68 Mg C ha^−1^ year^−1^ respectively. However, the carbon stock reduction in thinned stands compared to the stock of carbon in the unthinned plots was maintained over the entire 100 year period of observation.

**Conclusions:**

Thinning treatments in regenerating forest stands reduce forest carbon stocks, while carbon accretion rates recovered and were similar to unthinned stands. However, that the reduction of carbon stocks in thinned stands persisted for a century indicate that the unthinned treatment option is the optimal choice for short-term carbon sequestration. Other ecologically beneficial results of thinning may override the loss of carbon due to treatment. Our model estimates can be used to calculate regional carbon losses, alleviating uncertainty in calculating the carbon cost of the treatments.

**Electronic supplementary material:**

The online version of this article (doi:10.1186/s13021-015-0035-4) contains supplementary material, which is available to authorized users.

## Background

Forests play a key role in the global carbon cycle, containing an estimated 861 Pg C and providing a sink of 1.1 Pg C year^−1^ [1]. Forests are critical sinks for atmospheric greenhouse gases [2], and carbon fluxes occur across many carbon pools in forests, including live biomass, soils, and woody debris [3, 4]. The terrestrial carbon stock is generally stable over time scales of decades and can only slowly alter the total terrestrial carbon balance through gains or losses [4]. Disturbances that alter forest stands can provide dramatic departures from this characteristic pattern. An example is removal of carbon due to clearcut harvesting of forests, leading to a large loss of terrestrial carbon. The increase in biomass, or carbon accretion, as stands regenerate and grow after harvest is unknown in many forests. Thinning is a common silvicultural practice for increasing growth of individual trees and maintaining or increasing wildlife habitat. However, the influence of thinning on the carbon balance in young forests is uncertain in southeast Alaska. Carbon fluxes need to be evaluated across a range of management options to understand and estimate the short and long-term impacts of silvicultural treatments on carbon pools.

**Fig. 1:**
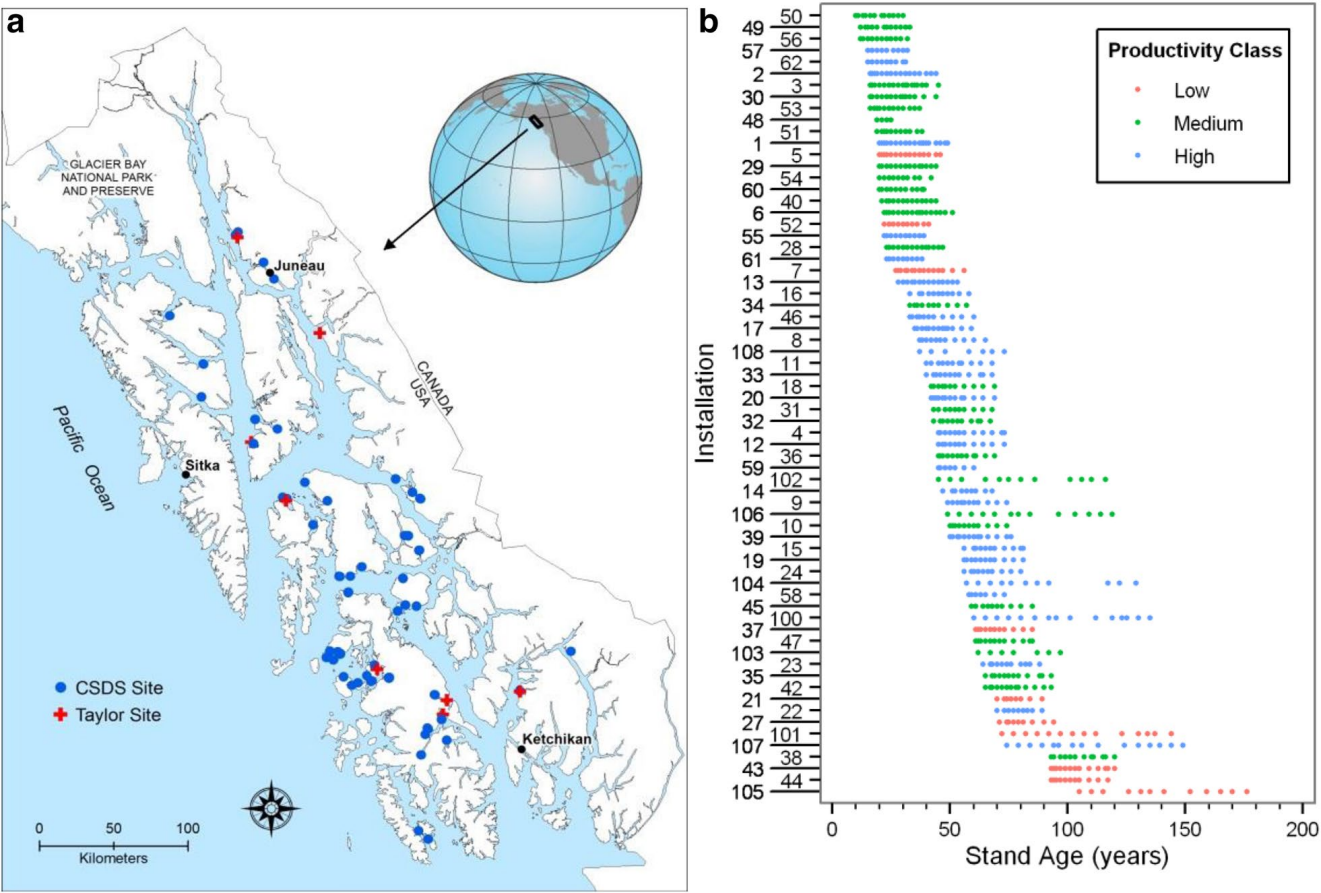
Locations of the 68 Farr and 12 Taylor installations in southeast Alaska (**a**). Each CSDS (“Farr”) installations consists of four plots: a control plot, a low-intensity thinned plot, a medium-intensity thinning plot, and a heavily-thinned plot. The Taylor installations consist of unthinned plots only and are generally in older stands. For a full description of the plots and thinning treatments see [19]. Data from the CSDS study arranged by age of stand at time of plot establishment (**b**). *Numbers* on Y axis refer to installation number with Farr plots <100 and Taylor plots ≥100. Productivity classes are the tertiles of the observed range for these sites as reported in [19]. Each *symbol* in an installation represents a measurement

Estimates of carbon flux in young-growth stands are needed to address land management planning goals and regional, national [5] and international carbon accounting protocols [6]. Mandates to understand the potential for forests to mitigate increasing concentrations of atmospheric CO_2_ require accurate accounting of forest carbon fluxes. The USDA Forest Service, for example, has priortized understanding carbon dynamics in forests as part of an overall strategy to protect the long-term health of forests [7]. Necessary information about carbon cycling is particularly lacking in the perhumid coastal temperate rainforests (PCTR) of the northeast pacific coastal margin [8] (Fig. 1).

Widespread commercial forest harvest has occurred across southeast Alaska for over 50 years. However, there is no estimate of the potential carbon sequestration across the ~452,000 ha [9] of young-growth forests in the region. Natural regeneration in PCTR forests is generally vigorous and leads to rapid and nearly complete occupation of space by conifer seedlings and saplings [10]. Densely-stocked stands can produce wood products similar to thinned stands [11], but the loss of light and density of overstory trees degrades the wildlife habitat [12, 13]. A common management intervention to alleviate the high stand density is thinning [14]. Felling of a portion of the stand basal area across a specific or variable [15] spacing can be applied to achieve maximum individual tree growth. However, thinning also alters the carbon accretion trajectory of the stand [4]. When left on site, the carbon content of thinned trees, and any trees that die naturally, can be accounted for by estimating decomposition rates. The impact of stand thinning and subsequent loss of biomass via decomposition are key components in calculationg a carbon sequestration rate for use in land management planning.

Quantifying the effects of young-growth forest management on carbon storage is challenging. Allometric equations linked to direct tree measurements can be used to estimate aboveground biomass production [16–18] across stand age, and this can be converted to carbon accretion. Estimation of the long-term differences between forests with varying management treatments requires remeasurement of the same plots over decades. Long-term plots provide an excellent source of information on biomass accretion over time where plots have been maintained and re-measured.

Experimental plots maintaned by the USDA Forest Service Pacific Northwest Research Station [19] offer an opportunity to estimate carbon change over time with varying levels of thinning. This dataset includes 284 plots across 68 installation sites, remeasured over several decades and spanning stand ages up to 161 years. The temporal and geographic breadth of these experimental plots provides an excellent foundation for investigating carbon standing stocks and carbon accretion rates in young-growth forests of the PCTR. In addition, the plot system allows analysis of the effects of forest thinning on carbon storage through the combination of allometric equations and repeated tree measurements over decades. We designed this study to address the critical need for an improved understanding of carbon storage in young-growth forest of the PCTR and to quantify the effects of thinning on carbon gain or loss. We hypothesized that while thinning may increase carbon accretion in individual trees, across whole stands thinning will have a neutral to negative impact on the sequestration of carbon, depending on the intensity of thinning.

## Methods overview

We utilized data from two long-term silvicultural datasets young-growth forests of southeast Alaska to estimate total tree carbon stock and accretion rate. One set of plots was started in the 1920’s and were not thinned (“Taylor plots”, 12 of 284). The other plot system included unthinned controls and thinning treatments applied at three intensities in a randomized block design (“Farr plots”, 272 of 284). Plot measurements included both live and dead trees, so estimates for both pools were calculated to account for the loss of dead tree carbon decomposing over time in both unthinned and treated forest stands. A new allometric model for small diameter trees was developed to fill a needed information gap in determination of carbon in small trees.

## Results

### Live and dead tree carbon pools in naturally-regenerating young-growth stands

**Fig. 2:**
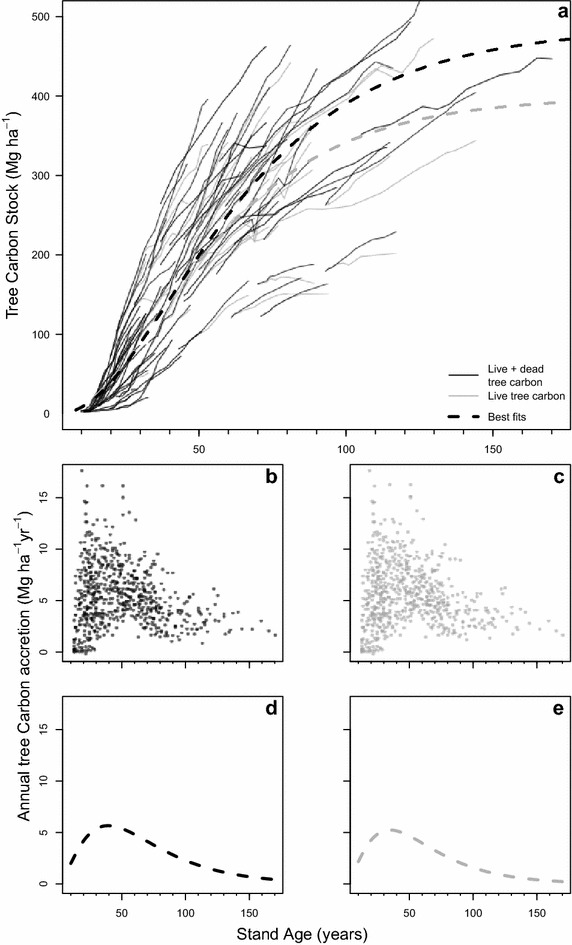
Carbon stock and carbon accretion in naturally-regenerating plots (Farr study control plots and Taylor plots). **a** Measured and modeled carbon stock through time. *Solid lines* describe carbon accretion measurements within individual plots across a range of ages. *Dashed lines* are NMLE best fit models for all tree carbon (live and dead trees) and for live tree carbon. Note that the plots with low carbon stock values are all located in the same sites which occurred on the lowest productivity areas that were sampled. Observed carbon accretion rates across stand age for **b** for all carbon (live and dead trees) and for **c** for live trees only. Implied carbon accretion (derivative of the NMLE model) as a function of stand age for **d** for all carbon (live and dead trees) and for **e** for live trees only

Live-tree carbon increased in unthinned young-growth stands across the stand age gradient and reached an asymptote of 398 (±20) Mg C ha^−1^ based on a best fit, non-linear mixed effect model (NLME) (Fig. 2a). The estimated asymptotic maximum carbon stock in the stands increased to 484 (±26) Mg C ha^−1^ with the inclusion of dead-tree carbon (Table 1) Dead trees in unthinned plots typically represent suppression mortality as tree density decreases through time. However, these mean carbon stock estimates for the measured plots have a great deal of uncertainty. A prediction interval was derived by considering observed variability within- and among-plots, in addition to the parameter uncertainty around the asymptote described above. The 90 % prediction intervals for the asymptotic carbon stock ranged from 145 to 653 Mg C ha^−1^ for the live-tree carbon model and 161–808 Mg C ha^−1^ for the model including both live- and dead-tree carbon.

**Table 1:** Parameter estimates (±SE) for best fit of carbon accretion in unthinned control stands for live tree only and for live + dead tree components of carbon using NMLE model

Model components	B_*0*_	B_*1*_	B_*2*_
Control (live + dead trees)	484.58 (25.90)	0.026 (0.0008)	0.636 (0.011)
Control (live trees only)	398.24 (20.03)	0.029 (0.0009)	0.634 (0.0119)

B_0_ is an estimate of the asymptote based on Eq. 3 where DBH is replaced by age and Mg C is calculated rather than height. B_*1*_ and B_*2*_ describe growth rate and the inflection point of the modeled relationship, respectively

We plotted carbon accretion as the change in the carbon pool over time in plots with only live tree carbon and calculated a peak at age 34.7 years (±0.5, bootstrap SE; Fig. 2b). The carbon accretion peaked at 39.3 years (±0.5, bootstrap SE; (Fig. 2c) for the model with both live and dead tree carbon. These carbon accretion rates varied dramatically across the chronosequence of measurements in the sampled stands (Fig. 2b, c). The high variability makes it difficult to estimate quantities with any reasonable level of precision directly from accretion data. While carbon accretion was more variable than carbon stock estimates, carbon accretion can also be estimated as the derivative of carbon stocks over time. The general shape of the data cloud suggests that accretion rates peak at 39 years and then decreases, tapering off at about 100 years. The shape of the accretion curves (Fig. 2d, e) derived directly from the fitted model for the total carbon stock (Fig. 2a) indicates that accretion peaks in young stands between 35 and 40 years and then tapers off as the stands age. The estimated weighted average carbon accretion rate based on the fitted model to total carbon [45] was 3.53 (±0.17) Mg C ha^−1^ year^−1^ for the live-tree carbon model and 3.81 (±0.20) Mg C ha^−1^ year^−1^ for the model that included live- and dead-tree carbon over the 150 years age span of measured trees.

### Influence of thinning on carbon accretion in young-growth stands

**Fig. 3:**
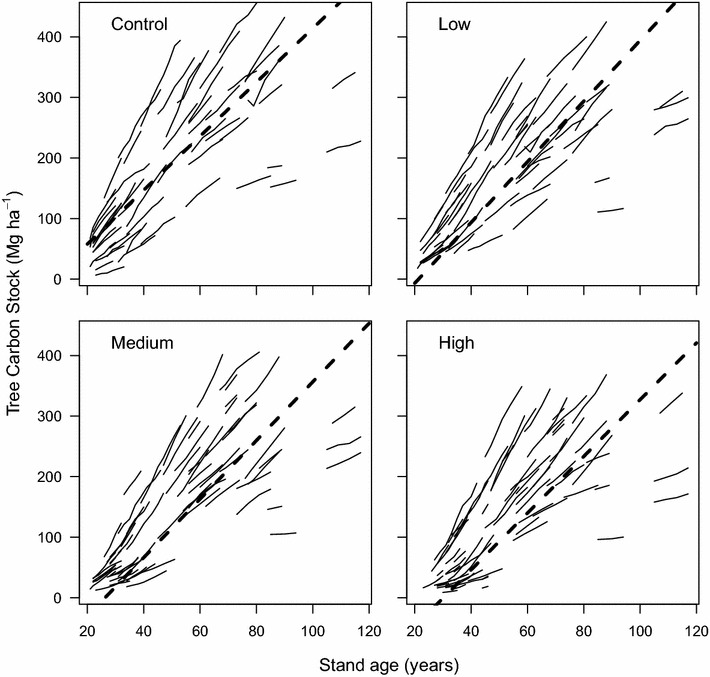
Modeled live tree carbon accretion 10-years post-thinning until end of study across treatment. *Dashed lines* are the estimated slopes for each line and describe accretion rates over time; however we note that much of the difference between treatments is accounted for in the intercept which describes live carbon stock 10 years post-thinning

**Fig. 4:**
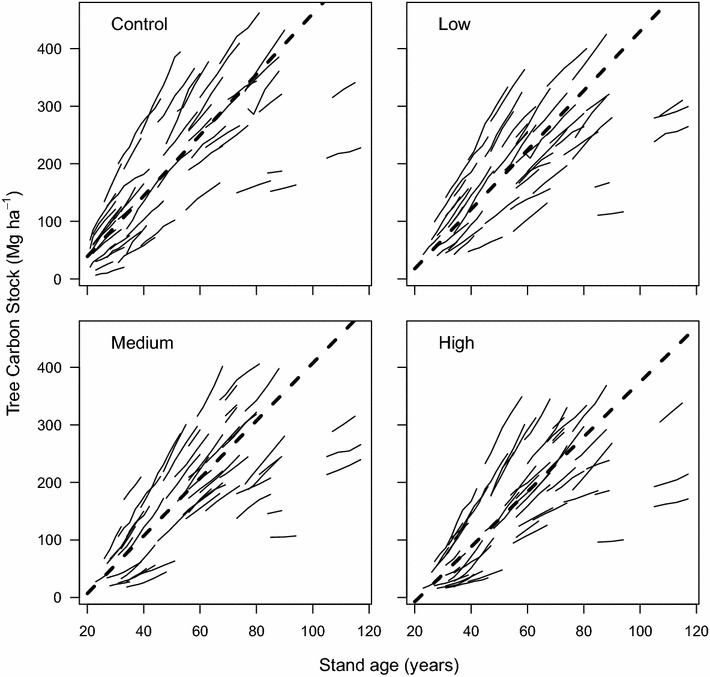
Modeled live and dead tree carbon accretion 10-years post-thinning until end of study across treatment types for the 164 of 215 study plots for which cut tree data were recorded. Dead tree carbon includes thinned trees and those that died naturally. *Dashed lines* are the estimated slopes for each line and describe accretion rates over time

There was a systematic decrease in the total stand carbon correlated with increasing intensity of thinning (Fig. 3). The portion of the carbon stock data in untreated young-growth stands that is nearly linear (20–100 years) was used as a basis for comparison between treated stands. The estimated average carbon pool in the unthinned control plots (Farr plots only, see methods) was greater than the estimated average carbon pool in any of the three thinning treatments at 40 years (Table 2); estimated average carbon pools at a given age consistently decreased with thinning intensity of treatments from low to high (Fig. 3; Table 2). The slope of the linear model fit to the data describes the stand-scale accretion rate. This accretion rate systematically decreased with thinning when decomposition of cut trees and any trees that died naturally is included and the total carbon stock was reduced by 16, 26, and 39 % across the low to high intensity thinning treatments at 40 years (Tables 2, 3). However, no major pattern between accretion rate and thinning intensity was noted with only live trees (Table 2; Fig. 4). We note that several plots, both control and treatment, displayed particularly low accretion rates and these plots were generally all located on one set of sites (Figs. 3, 4).

**Table 2:** Estimates of accretion rate and mean carbon density 40 y after thinning based on live tree carbon only

Treatment	Carbon accretion rate Mg C ha^−1^ y^−1^	Among plot standard error of accretion rate	Residual standard error	Carbon density at 40 y Mg C ha^−1^
Control	4.45 (0.31)	2.16	1.09	146.9 (7.4)
Low	5.01 (0.25)	1.87	1.28	93.4 (7.0)
Medium	4.83 (0.27)	2.20	1.04	66.3 (6.8)
Heavy	4.68 (0.31)	2.49	1.10	46.0 (6.2)

Low, medium, and heavy treatments refer to thinning intensity. Values in parentheses are parameter standard errors

**Table 3:** Estimates of accretion rate and mean carbon density 40 y after thinning based on live and dead tree carbon

Treatment	Carbon accretion rate (Mg C ha^−1^ y^−1^)	Among plot standard error of accretion rate	Residual standard error	Carbon density at 40 years (Mg C ha^−1^)
Control	5.27 (0.32)	2.26	0.93	144.3 (8.1)
Low	5.16 (0.28)	1.97	1.09	120.7 (6.9)
Medium	5.00 (0.31)	2.27	0.94	107.0 (6.8)
Heavy	4.78 (0.32)	2.39	1.15	88.1 (5.9)

These estimates are based on the 164 of 215 study plots for which cut tree data were recorded. Low, medium, and heavy treatments refer to thinning intensity. Values in parentheses are parameter standard errors

The residual model error in the linear models fit was similar across treatments for live trees using all plots (Tables 2, 3). This was also the case in models for live trees, cut trees, and natural mortality using plots for which cut tree data were available (221 of 284). This residual model error describes variability in carbon stocks within a plot over time after accounting for the effects of stand age and treatment. This standard error among plots for the accretion rate increased somewhat predictably across the three treatments suggesting that at more intensive levels of management it might be more difficult to predict accretion rates for an individual plot. Control plots were intermediate in their across-plot variability. We also note that residuals for both the live-tree and live-tree plus cut and natural dead tree models showed no trends over stand age, indicating that the linear model accurately described the underlying effect of stand age on carbon stocks, but residuals did show a somewhat increasing trend over chronological time indicating a potential increase in variability of carbon stocks in recent years.

### Simulation of stand carbon dynamics immediately after thinning

**Fig. 5:**
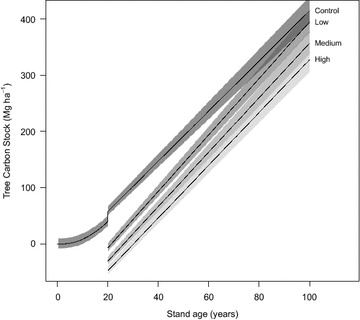
Scenario of live tree carbon trajectory for first 100 years of growth. Carbon accretion begins slowly, and then accelerates. This concave up curve comes from the non-linear model fit only to the control plots. When the stand reaches 20 years, we assume the plot was treated by thinning, and compare the carbon trajectories predicted by the four different treatment models. At 20 years, the treated plots immediately lose a large quantity of live carbon due to removal of woody material that was felled during the thinning from the live carbon pool. Ribbons cover the 50 % prediction interval, but do not include random effect variance among plots. The discontinuity in the control plots occurs because two separate models were used in this hypothetical scenario; the jump is due to the structural uncertainty in the models

We simulated a hypothetical carbon accretion scenario under different thinning intensities, all of which occur when stands reach 20 years of age, based on our fitted statistical models (Fig. 5). The simulated carbon stock at the plot scale accumulates at a rapidly accelerating pace in all plots until the stands are subjected to a simulated thinning at age 20. This thinning leads to the rapid drop in the carbon stock of thinned stands, as we only accounted for the carbon in the remaining live trees in the stands for the simulation. Stands in all four treatments begin accumulating carbon again after the thinning treatment is applied according to the linear models. We expect that increased growth rates of individual trees lead to a more rapid rate of carbon accretion after thinning on a per-tree basis. Note, however, that while individual trees may accrete carbon at a more rapid rate after thinning due to increased growth rates, there are many fewer trees accreting carbon in a thinned plot. Overall, at the plot scale, there is an initial loss of carbon in the thinned stands and a similar accretion rate to the control stands (Table 2). The simulated carbon stock in all thinning treatments remains lower than that of unthinned plots up to 100 years (Fig. 5).

## Discussion

### Carbon balance in unthinned forest stands

The rate and location of terrestrial carbon sinks is critical to understanding the global carbon balance. Young-growth forests sequester carbon in biomass, but at widely varying rates and over different timeframes. The calculation of total carbon stock and estimated accretion rates across the age gradient of the naturally regenerating young-growth forests of southeast Alaska fills a critical information gap for this region. The loss of live carbon after thinning in naturally-regenerating stands must be considered in calculating carbon sequestration estimates for young-growth forests. Thinning treatments are applied to achieve many ecosystem services in addition to carbon sequestration goals; therefore, our quantitative estimates of the loss of carbon after thinning enable evaluation of the carbon cost of a range of management actions for young-growth stand improvement.

Model calibration is essential for obtaining accurate carbon balance estimates across large regions [20]. Forest carbon models need to consider the entire range of stand types and ages to accurately portray the balance of carbon stock across the landscape [21]. Mature forest stands (>200 years) can accumulate carbon at an estimated 2.4 Mg C ha^−1^ year^−1^ [22]. The carbon stock in young-growth stands is particularly critical in these estimates as these stands are generally the most active zones of carbon change on the landscape due to rapid biomass accumulation and carbon storage in trees [23]. The estimated mean accretion rate of 3.53 Mg C ha^−1^ year^−1^ over 150 year in our study area confirms the strong net gain in carbon in young-growth stands in the Alaskan PCTR. This rate is higher than the 40 years mean of 2.71 Mg C ha^−1^ year^−1^ estimated in young-growth stands in the PCTR of British Columbia [24]. Frustratingly, the uncertainty in determining the response of an individual stand is high, which limits the usefulness of model predictions for site specific estimates of carbon stock, often needed to evaluate specific management scenarios. Our models are most appropriately applied across an entire population of stands for regional and national carbon assessments. Site-specific descriptors (e.g., site productivity) that might help stratify the data and provide more accurate predictions of carbon pools will need to be applied in order to help refine our predictions of carbon accretion rates in particular locations.

### Carbon balance in thinned young-growth stands

Maximizing the carbon stored in forests is a key goal of climate change mitigation programs [25]. The majority of the young-growth forest in the Alaskan PCTR result from harvest that occurred from 1960 to 1990 [9]. Thinning young-growth stands in the PCTR is a common management strategy to improve stand structure and wood production [14] and to improve wildlife forage production [13, 26]. Renewable energy recommendations for the Alaskan PCTR highlight the potential for wood energy projects using this stock of young-growth forest [27]. However, the usual management scenario for these young-growth stands is thinning at 15–20 years [13] and nearly half of the 25–50 year old stands have been pre-commercially thinned [9, 14]. Therefore, recognizing the tradeoff between thinning for stand improvement, biomass energy, and carbon sequestration in young-growth forest stands is important for making land management decisions. A key finding in our study is that thinning persistently reduces the carbon stock in young growth stands. The rate of carbon accretion in thinned stands is higher than control plots after the initial carbon loss; but, the gap created by the initial carbon loss is maintained and the total stock of carbon in thinned stands does not equal the stock of carbon in the control plots over the entire 100 year period of observation. This is consistent with the observation that the reduction in total stand carbon stock may not change the net ecosystem exchange between pre- and post-thinning [28]. The maintenance of tree growth would explain the similarity in the trajectory of carbon accretion among the treatments after the initial period of disturbance.

Reduced carbon stocks due to thinning have been recognized in other forests [4, 29, 30], but is not often included in forest carbon accounting or management actions due to the lack of adequate stand response data. Our quantification of the reduction in carbon stock across a range of thinning treatments allows estimates of the effects of thinning on regional carbon stocks. The systematic variation in the carbon stock related to thinning intensity may offer a mitigation measure for achieving benefits for wildlife, wood quality, or understory abundance and diversity in managed stands. The enhanced growth of understory plants after thinning represents a tradeoff of energy from trees to forest floor and a reduction in overstory carbon compared to unthinned stands. Benefits of thinning young growth need to be balanced with the desire to maximize carbon storage in forests. For example, the less intensive thinning treatments maintain more carbon, but still provide a benefit for other desired conditions in a stand. As demonstrated by our comparison, the unthinned option provides the greatest carbon accretion of all of the thinning prescription options.

### Limitations of analysis and information gaps

The carbon values provided in our study will be critical for estimating the carbon stock in the pool of young-growth forest in southeast Alaska, but, there is still considerable uncertainty in the range of carbon accretion values among the stands in our analysis. Therefore, site specific projects will need an improved model that is able to better reflect local conditions to carbon flux values. Factors that influence the variability in forest productivity among the sites or the response to thinning were included as random effects, but not specifically as predictive variables. Possible interactions with temperature [28], geology [31], soil saturation [32], nutrients [33] or other site-specific factors may play a role in site productivity. This uncertainty might be addressed by obtaining further information on the site factors that may influence the productivity of the plots such as soil, hydrology, or climate variables.

Potential alternate trajectories in the carbon accretion of thinned stands may arise that lead to different conclusions related to unthinned stands. We applied the same allometric equations to both unthinned and thinned stands in our analysis. It is possible that tree growth forms differ by thinning treatment and so biomass allocation would change in thinned stands. We are not aware of any existing allometric models for thinned stands of the PCTR. Therefore, we rely on the literature from other regions to support our conclusions and highlight that thinning has been found to primarily impact the biomass of the bole [34] and crown [35] of the thinned trees. Thinned stands can shift biomass accumulation from branch to leaf, but measured changes in bole biomass have been demonstrated to be small [36] unless very heavily thinned [37]. These observations provide some confidence that the total biomass calculated by our approach will not substantially change, but may be re-distributed within the tree after thinning.

The residual trees left after thinning grow at an accelerated rate, but these trees are generally left in a condition where they do not maximize the growing space for many years. Thinning goals such as increased individual tree growth and allocation of energy to the forest floor for plant diversity lead to lower overstory biomass accumulation in thinned plots. While the growth rate for individual trees is greater in these plots, the amount of biomass accumulation that would be required by the individual residual trees to match the loss in biomass of similar unthinned stands would be physiologically difficult to attain. The difference is illustrated in our evaluation of the stands at 40 years in Tables 2 and 3. There could be cases where a light thinning leaves a higher density than other thinning treatments, in which case, the thinned stand may accumulate biomass similar to unthinned stands due to the additional growth of the residual trees. However, this scenario is unlikely to be applied under most operational applications.

## Conclusions

Knowledge of the stock and rate of carbon accretion greatly enhances the understanding of carbon dynamics in the coastal forests of Alaska. The loss of carbon due to thinning can be used in the evaluation of management scenarios that address young-growth stand improvement. Regional carbon budgets will also be improved with estimates that include the carbon pool in young-growth stands of the PCTR.

## Methods

### Source of data

This study used data from the Cooperative Stand Density Study (CSDS; Fig. 1), comprised of two long-term silvilcultural field studies, previously compiled and published [19, 38, 39] and an earlier study implemented by Ray Taylor ("Taylor plots"; Fig. 1). Most data (272 of 284 plots) were from a study of thinning treatments on even-aged young-growth (<100 years) stands begun in 1974 and with remeasurements continuing until 2003 (“Farr plots”). The remaining 12 plots (“Taylor plots”) were located in older even-aged stands initiated by windthrow or early timber harvest in the late 19th century. The original intent of the studies was to measure sites that represented commercially harvested forests. Both the harvested landscapes and the plots in this study are weighted towards higher productivity classes. The Taylor plots were first measured in the late 1920s, with remeasurement occurring periodically through 2003. The Farr plots were established to examine growth and yield and how regenerating forest stands were impacted by light (mean 47.7 % BA removal); medium (mean 60.9 % BA removal); and heavy (mean 73.5 % BA removal), thinning at varying stand ages across varying productivity classes (Fig. 1). A complete description of thinning prescriptions is available in [19]. Most of these stands initiated following clear-cut harvest, with a smaller number of the older stands initiated by windthrow. All plots were dominated by western hemlock (*Tsuga heterophylla (Raf.) Sarg*) and Sitka spruce (*Picea sitchensis (Bong.) Carr*), with small amounts of western redcedar (*Thuja plicata*) and red alder (*Alnus rubra*). Stand age at thinning treatment ranged from 10 to 93 years (Fig. 2b). In general, the four treatments (control, light, medium, and heavy thinning) were applied in a randomized block design across 62 installations. Plot age, productivity class, and remeasurement dates are shown in Fig. 1b.

### Estimating biomass of live trees

Tree species and diameter at breast height (DBH) were recorded for each tree in the original study and at each remeasurement interval (roughly 2–5 years). A subset of trees (7308 of the 27562) was measured for height during each remeasurement using a clinometer and tape or laser. Tree heights were estimated from diameter and height relationships for the remaining trees (Additional file 1: Appendix A). DBH and height were used to estimate carbon using allometric equations of the form:

1 $$ B = b_{0} + b_{1} d^{2} h $$

where *d* is the diameter at breast height (DBH, in meters), *h* is the height above breast height (m), and *B* is the dry biomass (kg) for all the aboveground and belowground components of the tree [17]. The constant *b*_*0*_ is the biomass of a tree at breast height and *b*_*1*_ is related to the tree’s density. The constants *b*_*0*_ and *b*_*1*_ are species-specific. We separately accounted for red alder (*Alnus rubra* Bong.), Shore pine (*Pinus contorta* var. *contorta* Douglas ex. Loudon), western redcedar (*Thuja plicata* Donn ex D. Don), Sitka spruce (*Picea sitchensis*) and western hemlock (*Tsuga heterophylla*). Calculations for any other species were done with the western hemlock equations from [17]. Note that Sitka spruce and western hemlock account for more than 98 % of all tree measurements.

The equations developed by Standish et al., [19] had a minimum tree diameter of 3.1 to 5.3 cm, and due to the large intercept terms, did not accurately estimate the biomass of small trees. The presence of many small diameter trees in our database required the development of a new equations We developed allometirc biomass equations for small trees by sampling 60 small diameter Sitka spruce and western hemlock and calculating the total biomass based on whole tree harvest and weighing (Additional file 2: Appendix B). These empirical biomass relationships for small diameter trees were based on Sitka spruce and western hemlock trees (<7.5 cm dbh) sampled in three locations arrayed across the geographic region of the database (Additional file 2: Appendix B). The dbh threshold for using our empirical biomass estimates for small trees versus the constants from Standish et al. [19], suitable for larger trees, was defined by the intersection of our local parameterization curve and the Standish parameterization under the assumed height-diameter relationship (Additional file 2: Appendix B). Because the height-diameter relationship and allometric parameterizations were species-specific, the diameter threshold that determined which biomass equation to apply was also unique to each species.

### Estimating biomass of dead trees

Dead trees, both those cut during thinning and left on site and those that died from natural mortality, are often ignored in estimates of forest carbon pools and fluxes. In our analysis all cut trees were considered to be left on site to decompose. Cut trees were recorded in 164 of 215 treatment plots. Most plots missing cut tree data were reported in [19] as lacking pre-thinning data. The exceptions are the 16 treatment plots of installation 62 (“Staney Creek”), for which no explanation of the missing cut tree data is given. In all cases, analysis that considered the effect of management on dead trees was based on the 164 plots for which cut tree data were available.

We estimated carbon content of dead trees using the following deterministic relationship previously parameterized for the region in a study of the decomposition rate of thinning slash [40]:

2 $$ \begin{aligned}   B &= B_{0} [0.3870\exp ( - 0.1429\;t) \hfill \\   &  \quad  +0.6198\exp ( - 0.00223\;t)] \end{aligned}  $$

where *B*_*0*_ is the estimated biomass at the time of death in kilograms, and *t* is time since tree death in years. This equation was used for both trees that were cut at the beginning of the study during initial thinning and left on site as well as for trees that died of natural causes, typically from suppression, at some point during the study’s duration. For the latter case, we assumed the tree died and began decomposition at the midpoint between the date on which the last live measurement was taken and the date on which it was marked as dead.

### Estimating carbon at the plot level from individual tree biomass

We assumed that carbon made up 48 % of the dry biomass [41] of an individual tree for both live and dead trees and that the root to aboveground biomass ratio was 0.2 [17]. Carbon estimates over all trees within a plot were aggregated into a single estimate of megagrams of carbon per hectare.

### Ingrowth

Due to irregular inclusion of ingrowth measurements, our analysis of carbon estimates did not account for biomass additions due to ingrowth of new trees. We evaluated the potential impact of excluding ingrowth in our carbon estimates for plots with available ingrowth measurements. In 95 % of the measurements, the contribution of ingrowth was <5 % of total plot carbon. However, the error from excluding ingrowth likely increases with stand age as these forests begin to reach the understory re-initiation phase [42].

### How does carbon accretion change with stand age in naturally-regenerating forests?

To understand basic underlying carbon dynamics of young growth stands in the PCTR, we first evaluated naturally-regenerating plots. By combining data from the Farr control plots and the Taylor plots, none of which were thinned, we had a very long chronosequence of naturally-regenerating plots (Fig. 1b), measured between 1926 and 2000 that were 10 to 170 years of age. We fit an asymptotic nonlinear equation to relate carbon content to stand age [43]:

3 $$ TC = A\left[ {1 - \exp \left( {b_{1} \;age} \right)} \right]^{{\frac{1}{{1 - b_{2} }}}} , $$

where TC is total carbon in a stand (Mg ha^−1^) and stand age (*age*) is measured in years. We used non-linear mixed effects models to account for correlation among repeated measures within plots, thereby allowing the stand index to implicitly enter the model as a random feature of each plot. The random effect was placed on the asymptotic amount of carbon in the plot, consistent with the idea that the random effect reflects differences in site productivity index. Models were fit using the nlme package in R [44]. The model was first fit using estimates of carbon from live trees only. We then fit the model again to estimate carbon based on both live and dead trees.

We estimated the weighted average rate of carbon accretion as:

$$ \frac{{Ab_{1} }}{{2b_{2} + 2}} $$

Using this equation [45], we weight the instantaneous rate of accretion, so that the steeper portion the curve, is most influential when accounting for overall carbon. Estimates of parameter uncertainty were derived using parametric bootstrapping.

### How does carbon accretion change with thinning?

We examined the impact of the three thinning treatments on carbon accretion using the 272 Farr plots. We did not include data from the Taylor plots in this analysis as there were no equivalent examples of older thinned plots. Carbon dynamics in the first 10 years after thinning were nonlinear due to the rapidly deccelerating pace of decomposition of cut trees. These early data describe a different ecological process than data from >10 years post-thinning and were therefore excluded from our model. We excluded the first 10 years of measurements from control plots in the same blocks to balance the design. Within this age range of approximately 20–100 year-old stands, the carbon stock increased linearly among all four treatments. Therefore, we fit a linear mixed effects model to this data set. A random effect was placed on both the intercept and the slope, which was supported by likelihood ratio test, P < 0.001. These slopes describe the estimated average carbon accretion rate for stands within each treatment.

## Additional files

**Additional file 1: Appendix A.** Estimating tree height.

**Additional file 2: Appendix B.** Estimating biomass for small trees.
