# Supplementary material for: Carbon accretion in unthinned and thinned young-growth forest stands of the Alaskan perhumid coastal temperate rainforest
Source: Carbon Balance Manag. 2015 Oct 20;10:25. doi: 10.1186/s13021-015-0035-4 (PMC4610996; doi:10.1186/s13021-015-0035-4)
Supplement: Supplementary file 1 — Additional file 1: Appendix A. Estimating tree height. [file 13021_2015_35_MOESM1_ESM.docx]

Appendix A: Estimating tree height

Equation (1) requires an input of height, in addition to DBH. During data collection height was measured for a subset of trees in each plot to establish site productivity and tree height-diameter relationships. In the original database, 7308 of 27562 total trees had at least one height measurement, leaving roughly 73% of trees without height measurements. For trees with no or intermittant height measurements we estimated heights by the following procedure. For trees with at least five height measurements, we predicted any heights in sampling where they were not measured using a linear regression between DBH and height for that individual tree. If fewer than five height measurements existed, we predicted remaining heights using a regression between DBH and height for all measurements of a given species. For *T. heterophylla* and *P. sitchensis*, this was a non-linear regression of the form:

$h=A\left[ 1-\exp\left( b_{1}d \right) \right]^{\frac{1}{1-b_{2}}}$ (2)

where *h* is height, *d* is DBH, *A* is the asymptotic height for that species, *b_1_* is a growth rate parameter and *b_2_* controls at what height the inflection point of the relationship occurs [1, 2]. This relationship was estimated separately for each treatment (control, and high, medium, and low intensity thinning) because we expect the treatment to impact the relationship between DBH and tree height. For remaining species (*T. plicata, A. rubra, P. contorta*), the model including all available height measurements was a simple linear regression, like the individual tree model, as the smaller sample sizes available did not provide sufficient contrast for an asymptotic model. The *T. plicata* relationship was estimated separately for each treatment; because of small sample sizes, the remaining two relationships were not.

[1] Richards FJ. A flexible growth function for empirical use. Journal of experimental botany. 1959;10:290-301.

[2] Melson SL, Harmon ME, Fried JS, Domingo JB. Estimates of live-tree carbon stores in the Pacific Northwest are sensitive to model selection. Carbon balance and management 2011;6:1-16.
