# Supplementary material for: Carbon accretion in unthinned and thinned young-growth forest stands of the Alaskan perhumid coastal temperate rainforest
Source: Carbon Balance Manag. 2015 Oct 20;10:25. doi: 10.1186/s13021-015-0035-4 (PMC4610996; doi:10.1186/s13021-015-0035-4)
Supplement: Supplementary file 2 — Additional file 2: Appendix B. Estimating biomass for small trees. [file 13021_2015_35_MOESM2_ESM.docx]

Appendix B: Estimating biomass for small trees

Closer examination of the Standish et al. (1985) equations indicated that they significantly over-estimate the biomass of small trees because *b_0_* is not an accurate estimate of the biomass of a tree at breast height. Therefore, we collected, dried, and weighed young *T. heterophylla* and *P. sitchensis* and re-parameterized these equations using the same form (Table B1). Analysis of the data for small trees indicated no significant difference between a single model and separate *b_0_* and *b_1_* for the two species (*F* = 1.27, df = 2, *P* = 0.2872). Therefore, we aggregated small tree data for both species into a single model that was used for all four conifer species. We had not found an over-estimation problem for small *A. rubra*; as a deciduous tree, it may have a different relationship between biomass and DBH and height, so we did not used the newly parameterized equation based on the young *T. heterophylla* and *P. sitchensis* for *A. rubra*. In deciding whether to use the small conifer equation or the species-specific large tree regresssion for estimating biomass in conifers, we found the DBH at which the two curves intersected, assuming that height and DBH followed the estimated relationship for that species. The DBH at the intersection was our cutoff; for all species it falls between 5 and 10 cm.

| Table B1. Small diameter tree database used in development of estimates of biomass. | | | | | | |
| --- | --- | --- | --- | --- | --- | --- |
| Site | TreeNumber | Species | DSH_cm* | DBH_cm** | Height_cm | TotalDryWt_g |
| Juneau | 1 | Tshe | 2.6 | 0 | 137 | 440.0 |
| Juneau | 2 | Pisi | 4.7 | 3.2 | 237 | 2655.0 |
| Juneau | 3 | Pisi | 3.5 | 2.5 | 249 | 1075.0 |
| Juneau | 4 | Tshe | 3 | 2.3 | 237 | 860.0 |
| Juneau | 5 | Tshe | 5.3 | 3.8 | 292 | 3325.0 |
| Juneau | 6 | Pisi | 7.2 | 4.9 | 369 | 5725.0 |
| Juneau | 7 | Pisi | 3.4 | 2.1 | 191 | 1110.0 |
| Juneau | 8 | Pisi | 5 | 2.5 | 212 | 1815.0 |
| Juneau | 9 | Pisi | 6.2 | 4.3 | 358 | 3685.0 |
| Juneau | 10 | Pisi | 4.3 | 1.9 | 176 | 1035.0 |
| Juneau | 11 | Pisi | 5.5 | 3.4 | 264 | 2095.0 |
| Juneau | 12 | Pisi | 2.5 | 0.3 | 137 | 455.0 |
| Juneau | 13 | Pisi | 3.2 | 1.5 | 188 | 665.0 |
| Juneau | 14 | Pisi | 5.7 | 4 | 319 | 3190.0 |
| Juneau | 15 | Pisi | 4.1 | 3 | 293 | 1855.0 |
| Juneau | 16 | Tshe | 3.9 | 1.6 | 244 | 1170.0 |
| Juneau | 17 | Tshe | 2.9 | 2.1 | 199 | 900.0 |
| Juneau | 18 | Tshe | 2.3 | 1.2 | 188 | 470.0 |
| Juneau | 19 | Tshe | 1.7 | 0.2 | 151 | 205.0 |
| Juneau | 20 | Pisi | 2.7 | 1 | 189 | 465.0 |
| Juneau | 21 | Tshe | 3.2 | 0.3 | 195 | 160.0 |
| Juneau | 22 | Tshe | nd | 3.1 | 235 | 2525.0 |
| Juneau | 23 | Pisi | 2.2 | 0.2 | 142 | 295.0 |
| Juneau | 24 | Pisi | 1.9 | 0.3 | 138 | 280.0 |
| Juneau | 25 | Tshe | 6 | 3.9 | 390 | 3750.0 |
| Juneau | 26 | Tshe | 4.5 | 3.4 | 321 | 2780.0 |
| Juneau | 27 | Tshe | 6.4 | 4.1 | 401 | 4910.0 |
| Juneau | 28 | Tshe | 6.7 | 4.6 | 405 | 5740.0 |
| Kruzof | 29 | Tshe | 4.8 | 1.2 | 192 | 595.0 |
| Kruzof | 30 | Pisi | 3 | 2.2 | 317 | 900.0 |
| Kruzof | 31 | Pisi | 4.9 | 3 | 235 | 2225.0 |
| Kruzof | 32 | Tshe | 5.4 | 3.9 | 334 | 2500.0 |
| Kruzof | 33 | Tshe | 3.9 | 0.7 | 159 | 1110.0 |
| Kruzof | 34 | Tshe | 3.2 | 1.2 | 192 | 760.0 |
| Kruzof | 35 | Pisi | 3.3 | 1.5 | 186 | 1060.0 |
| Kruzof | 36 | Tshe | 4.7 | 1.7 | 203 | 1640.0 |
| Kruzof | 37 | Pisi | 2.8 | 0.1 | 137 | 470.0 |
| Kruzof | 38 | Tshe | 5.2 | 2.8 | 348 | 2600.0 |
| Kruzof | 39 | Tshe | 6.8 | 4.3 | 339 | 6515.0 |
| Kruzof | 40 | Pisi | 3.7 | 2.5 | 287 | 1275.0 |
| Kruzof | 41 | Pisi | 4.1 | 2.7 | 335 | 1650.0 |
| Kruzof | 42 | Tshe | 3.5 | 1.7 | 244 | 675.0 |
| Kruzof | 43 | Tshe | 5.2 | 3.6 | 248 | 2425.0 |
| Prince of Wales | 44 | Pisi | 3.7 | 0.1 | 140 | 899.8 |
| Prince of Wales | 45 | Tshe | 3.5 | 1.5 | 245 | 938.4 |
| Prince of Wales | 46 | Tshe | 3.2 | 1.4 | 290 | 815.7 |
| Prince of Wales | 47 | Tshe | 3.1 | 1.8 | 236 | 486.1 |
| Prince of Wales | 48 | Pisi | 5.7 | 2.4 | 217 | 1720.6 |
| Prince of Wales | 49 | Pisi | 3.5 | 0.9 | 180 | 768.7 |
| Prince of Wales | 50 | Pisi | 3 | 1.5 | 177 | 782.8 |
| Prince of Wales | 51 | Pisi | 3.7 | 1.2 | 166 | 632.3 |
| Prince of Wales | 52 | Tshe | 2.5 | 0.1 | 172 | 426.6 |
| Prince of Wales | 53 | Tshe | 5.9 | 3.8 | 434 | 3868.0 |
| Prince of Wales | 54 | Tshe | 9.1 | 5.7 | 455 | 10691.8 |
| Prince of Wales | 55 | Tshe | 4.5 | 2.1 | 245 | 706.5 |
| Prince of Wales | 56 | Tshe | 4.6 | 1.5 | 220 | 1116.8 |
| Prince of Wales | 57 | Pisi | 3.8 | 0.9 | 158 | 1243.2 |
| Prince of Wales | 58 | Pisi | 6.5 | 3.1 | 299 | 4425.0 |
| Prince of Wales | 59 | Pisi | 6.4 | 4.3 | 391 | 5452.5 |

*DSH-Diameter stump height (0.30 m); **DBH-Diameter breast height (1.37 m);
